# Evidence of Stage- and Age-Related Heterogeneity of Non-HLA SNPs and Risk of Islet Autoimmunity and Type 1 Diabetes: The Diabetes Autoimmunity Study in the Young

**DOI:** 10.1155/2013/417657

**Published:** 2013-12-04

**Authors:** Brittni N. Frederiksen, Andrea K. Steck, Miranda Kroehl, Molly M. Lamb, Randall Wong, Marian Rewers, Jill M. Norris

**Affiliations:** ^1^Colorado School of Public Health, University of Colorado, 13001 E. 17th Place, Box B119, Aurora, CO 80045, USA; ^2^Barbara Davis Center for Childhood Diabetes, University of Colorado, 1775 Aurora Ct., Aurora, CO 80045, USA

## Abstract

Previously, we examined 20 non-HLA SNPs for association with islet autoimmunity (IA) and/or progression to type 1 diabetes (T1D). Our objective was to investigate fourteen additional non-HLA T1D candidate SNPs for stage- and age-related heterogeneity in the etiology of T1D. Of 1634 non-Hispanic white DAISY children genotyped, 132 developed IA (positive for GAD, insulin, or IA-2 autoantibodies at two or more consecutive visits); 50 IA positive children progressed to T1D. Cox regression was used to analyze risk of IA and progression to T1D in IA positive children. Restricted cubic splines were used to model SNPs when there was evidence that risk was not constant with age. *C1QTNF6* (rs229541) predicted increased IA risk (HR: 1.57, CI: 1.20–2.05) but not progression to T1D (HR: 1.13, CI: 0.75–1.71). SNP (rs10517086) appears to exhibit an age-related effect on risk of IA, with increased risk before age 2 years (age 2 HR: 1.67, CI: 1.08–2.56) but not older ages (age 4 HR: 0.84, CI: 0.43–1.62). *C1QTNF6* (rs229541), SNP (rs10517086), and *UBASH3A* (rs3788013) were associated with development of T1D. This prospective investigation of non-HLA T1D candidate loci shows that some SNPs may exhibit stage- and age-related heterogeneity in the etiology of T1D.

## 1. Introduction

Type 1 diabetes (T1D) is a chronic autoimmune disease in which the insulin-producing beta cells of the pancreas are destroyed. There is typically a preclinical phase of circulating autoantibodies, called islet autoimmunity (IA), which precedes the clinical diagnosis of T1D. T1D is widely believed to be caused by an environmental factor on a susceptible genetic background. The major susceptibility locus for T1D maps to the HLA class II genes at chromosome 6p21. These HLA class II alleles account for 30–50% of the familial clustering of T1D [1].

More than 50 non-HLA T1D susceptibility gene markers have been confirmed. The major non-HLA loci include *INS *[2], *CTLA4* [3], *PTPN22* [4], *IL2RA* [5], and *IFIH1* [6]. The DAISY study has previously investigated 20 non-HLA SNPs and found SNPs in *PTPN22*, *UBASH3A*, *INS*, and *IFIH1* associated with IA and/or progression to T1D [7–11].

Prospective birth cohorts have the unique ability to study two stages in the natural history of T1D: development of IA and progression to T1D in IA positive children. Different exposures have been associated with one or both stages. For instance, DAISY recently identified an association between a gene-gene interaction involving the vitamin D receptor gene (*VDR*) and protein tyrosine phosphatase, nonreceptor type 2 gene (*PTPN2*) with progression to T1D in IA positive children, but not with development of IA [12]. This would be an example of stage-related heterogeneity in the natural history of T1D. There is also evidence of age-related heterogeneity in the etiology of T1D when a gene or exposure is associated with the disease at certain ages, but not others. One example is a recent study that found differences in metabolite profiles relative to age, in which there was an association between lower methionine levels and presence of diabetes autoantibodies in younger onset (≤2 years), but not older onset (≥8 years) autoimmunity [13]. The purpose of this analysis was to investigate stage- and age-related heterogeneity of fourteen non-HLA T1D candidate SNPs for their association with development of IA and progression to T1D in a prospective birth cohort of non-Hispanic white (NHW) children at increased genetic risk of T1D. Additionally, we investigated whether the fourteen T1D candidate SNPs that were originally detected by GWAS using a case-control study design would be detected in time-to-event analyses of T1D risk in a prospective birth cohort.

## 2. Materials and Methods

### 2.1. Subjects

The Diabetes Autoimmunity Study in the Young (DAISY) is a prospective study composed of two groups of children at increased risk for T1D who were recruited between 1993 and 2004 and are being followed prospectively for the development of IA and T1D. One group is made up of first degree relatives of patients with T1D, identified and recruited between birth and eight years of age, mainly through the Barbara Davis Center for Childhood Diabetes (*n* = 815). The second group consists of infants born at St. Joseph's Hospital in Denver, CO, whose umbilical cord blood was screened for diabetes-susceptibility HLA-DR, DQ genotypes (*n* = 819). Details of the newborn screening, sibling and offspring recruitment, and followup of both cohorts have been published previously [14, 15]. Cord blood or the first available blood sample (depending on enrollment group) was sent to Roche Molecular Systems, Inc., Alameda, CA, for PCR-based HLA-DR, DQ typing. All study protocols were approved by the Colorado Multiple Institutional Review Board, and informed consent was given by parents of all participating children.

### 2.2. Measurement of Autoantibodies

Autoantibodies were tested at 9, 15, and 24 months, and annually thereafter, or at their first visit and annually thereafter if the child enrolled after birth. Radioimmunoassays were used to measure serum autoantibodies to insulin, GAD-65, and IA-2 (BDC512), as previously described [16–19], with rigorous confirmation of all positive and a subset of negative results. The cut-off for positivity was established as the 99th percentile of healthy controls. Children who tested autoantibody positive were put on an accelerated testing schedule of every 3–6 months.

Cases of IA were defined as those children positive for at least one islet autoantibody (IAA, GAD-65, IA-2) on two or more consecutive visits. T1D was diagnosed by a physician and defined as random blood glucose >200 mg/dL and/or HbA1c (A1C) >6.5% with clinical symptoms of T1D.

### 2.3. Non-HLA SNP Genotyping

DAISY children were genotyped for fourteen non-HLA T1D candidate SNPs: *C1QTNF6* (rs229541), *C6orf173* (rs9388489), *C14orf181* (rs1465788), *IL2* (rs2069762), *IL2* (rs4505848), *IL2RA* (rs12722563), *IL2RA* (rs2104286), *IL7R* (rs6897932), *PRKCQ* (rs947474), *SKAP2 *(rs7804356), *SMARCE1* (rs7221109), *TLR8* (rs5979785), *UBASH3A* (rs3788013), and SNP (rs10517086). Thirteen of the fourteen SNPs were chosen from three GWAS meta-analyses [20–22]. *UBASH3A* (rs3788013) was chosen based on its strong LD with *UBASH3A* (rs876498), which was discovered for its association with T1D by Concannon et al. [23].

The SNPs were genotyped utilizing the Taqman SNP genotype based OpenArray platform (Applied Biosystems, CA, USA). Custom designed 48-sample arrays and normalized genomic DNA were loaded using the OpenArray AccuFill system and cycling was performed on a GeneAmp 9700 PCR system (Applied Biosystems, CA, USA), all according to manufacturer protocol. Alleles were analyzed using the OpenArray SNP genotyping analysis software v.1.0.3 and Taqman Genotyper Software 2.0 (Applied Biosystems, CA, USA). All fourteen SNPs had a 95% call rate or higher.

Each SNP was tested for consistency with Hardy-Weinberg proportions using a 1-degree of freedom *χ* ^2^ goodness-of-fit test with a *P* value of 0.05 considered as evidence of a departure from Hardy-Weinberg equilibrium; all fourteen SNPs were in Hardy-Weinberg equilibrium. Linkage disequilibrium (LD) was tested in our population using Haploview version 4.2 as measured by *r* ^2^ and *D'*, with *r* ^2^ = 0.257 and *D'* = 0.862 for the two *IL2RA* SNPs and *r* ^2^ = 0.222 and *D'* = 1.0 for the two *IL2* SNPs.

### 2.4. Analysis Population

We obtained genetic data on at least one of the fourteen non-HLA T1D candidate SNPs for 1634 non-Hispanic white children in the DAISY cohort. This included 132 children who developed IA, of whom 50 went on to develop T1D. Fifteen IA cases were positive for autoantibodies on their first clinic visits; these left-censored cases were removed from the development of IA analysis cohort but retained in the progression from IA to T1D cohort. The same 50 IA positive children who went on to develop T1D are the same 50 T1D cases in the development of T1D analyses. However, not all IA positive children went on to develop T1D. All statistical analyses were limited to non-Hispanic whites in the DAISY cohort.

### 2.5. Statistical Analyses

SAS version 9.3 (SAS Institute Inc., Cary, NC, USA) statistical software package was used for all statistical analyses. SNPs were tested for violation of the proportional hazards assumption using a supremum test, with a *P* value < 0.20 indicating possible departure from proportional hazards [24]. If a SNP appeared not to meet this assumption, restricted cubic splines were used to evaluate the nature and extent of the violation. SNPs were analyzed for their association both with development of IA and with progression from IA to T1D. For each model, hazard ratios (HR) and 95% confidence intervals (CI) were estimated using Cox regression analyses. There were 302 sibling pairs in the analysis cohort, so a clustered time to event analysis was performed treating siblings from the same family as a cluster, and robust sandwich variance estimates were used for statistical inference [25]. Analyses of time to development of IA were adjusted for the HLA-DR genotype (HLA-DR3/4, DQB1*0302 versus other genotypes) and presence of a first degree relative with T1D. Analyses of time to progression to T1D were adjusted, in addition, for age at first positive autoantibody visit. *TLR8* (rs5979785) was additionally adjusted for sex because it is on the X chromosome. The significance threshold was defined as *α* = 0.05. Because our analyses were based on *a priori* hypotheses with SNPs previously found to be associated with T1D, *P* values were not corrected for multiple testing. We analyzed each non-HLA SNP in separate, covariate adjusted models. In the analyses examining development of IA, all of the SNPs were treated additively, except *IL2RA* (rs12722563) and *PRKCQ* (rs947474), which were dichotomized on the minor allele due to small sample sizes. Additionally, in the progression to T1D analyses, SNPs were treated additively, except *C14orf181* (rs1465788), *IL2RA* (rs12722563), *IL2RA* (rs2104286), *IL7R* (rs6897932), *PRKCQ* (rs947474), *SKAP2* (rs7804356), and SNP (rs10517086), which were dichotomized on the minor allele due to small sample sizes.

## 3. Results

### 3.1. Development of IA

We first examined whether non-HLA variants were associated with development of IA. The mean age at first IA positive visit was 6.2 years, and the mean age at last follow-up visit in children who did not develop IA was 9.9 years (Table 1). IA positive children were more likely to have the HLA-DR3/4, DQB1*0302 genotype compared to DAISY children who did not develop IA (HR: 2.97, 95% CI: 2.01, 4.39).

**Figure 1:**
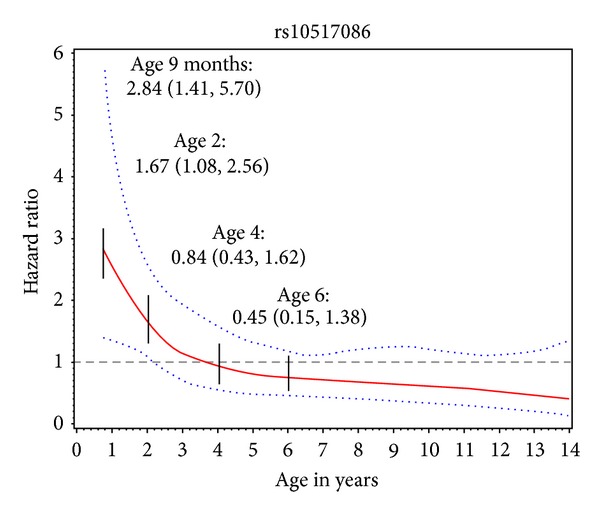
Association between SNP rs10517086 and development of IA modeled using a restricted cubic spline. The hazard ratio at different ages is represented by the solid line and the 95% confidence intervals are represented by the dotted lines. Hazard ratios and 95% confidence intervals are shown at 9 months, 2 years, 4 years, and 6 years (denoted with short vertical lines) illustrating the increased risk of developing IA in the younger ages, but not in older ages.

Unadjusted SNP association analyses are presented in (see Supplemental Table 1 in Supplemetary Material available online at http://dx.doi.org/10.1155/2013/417657). Adjusting for HLA-DR3/4, DQB1*0302 and first degree relative with T1D, *C1QTNF6 *(rs229541) was associated with development of IA (HR: 1.57, 95% CI: 1.20, 2.05 (for each additional minor allele)) (Table 2). SNP (rs10517086) did not appear to meet the assumptions of proportional hazards in the development of IA analysis and therefore was modeled using a restricted cubic spline. The restricted cubic spline shows that SNP (rs10517086) is associated with an increased risk of developing IA before the age of two or in younger ages but is not associated with developing IA in older ages (Figure 1). Children with a minor allele developed IA significantly earlier than children with no minor alleles (mean age at onset of IA: 7.2, 5.2, and 3.2 for 0, 1, and 2 minor alleles, resp., *P* = 0.003).

### 3.2. Progression to T1D in Children with IA

We then examined whether non-HLA variants were associated with progression to T1D in IA positive children. Of the 132 IA positive children in DAISY, 50 developed T1D; the mean age at T1D diagnosis was 8.7 years (Table 1). The mean age at last followup visit in nondiabetic children with IA was 14.1 years. Children who developed T1D were younger when they first tested positive for an autoantibody than IA positive children who have not progressed to T1D, 3.9 and 7.2 years, respectively (*P* = 0.003). Children with IA who developed T1D were more likely to have the HLA-DR3/4, DQB1*0302 genotype compared to children with IA who did not progress to T1D (HR: 2.79, 95% CI: 1.65, 4.72). Adjusting for HLA-DR3/4, DQB1*0302, first degree relative with T1D, and age at first IA positive visit, none of the fourteen non-HLA T1D candidate SNPs was associated with progression to T1D in IA positive children (Table 2). SNP association analyses adjusted only for age at first IA positive visit are presented in Supplemental Table 1.

### 3.3. Development of T1D

In order to evaluate the same outcome as previous GWAS to see if similar associations could be seen using a time-to-event analysis in a prospective birth cohort and to better understand the role these SNPs play in the natural history of T1D, we examined whether these non-HLA variants were associated with T1D in our population of 1619 children. All 50 children who developed T1D had developed IA previously, so the same 50 T1D cases were included in both the progression from IA to T1D (presented in Section 3.2) and these development of T1D analyses. However, not all IA positive children went on to develop T1D during followup. Unadjusted SNP association analyses are presented in Supplemental Table 1. Adjusting for HLA-DR3/4, DQB1*0302 and first degree relative with T1D, three of the fourteen non-HLA T1D candidate SNPs were associated with development of T1D (Table 2). Two of the SNPs associated with development of T1D, *C1QTNF6* (rs229541) and SNP (rs10517086), were also associated with development of IA, but not with progression to T1D in IA positive children. The other SNP associated with development of T1D, *UBASH3A* (rs3788013), was not associated with development of IA nor progression to T1D in IA positive children.

## 4. Discussion

In exploring associations between fourteen previously discovered non-HLA T1D candidate SNPs and the development of IA and progression to T1D in the prospective DAISY cohort, we found that *C1QTNF6* (rs229541) predicts IA but not progression to T1D, demonstrating stage-related heterogeneity. Moreover, SNP (rs10517086) demonstrates age-related heterogeneity with predicting IA only in the youngest ages. These two SNPs were also associated with development of T1D in our cohort, as well as *UBASH3A* (rs3788013). It is possible that the observed associations between both *C1QTNF6* (rs229541) and SNP (rs10517086) and development of T1D were driven by their association with development of IA. Given that all of our T1D cases developed IA prior to clinical diagnosis, it was not possible to determine whether a gene was associated with T1D via a pathway other than IA.

*C1QTNF6* (rs229541), which was first identified through a meta-analysis of data from three genome-wide association studies (GWAS) (combined *P*  value = 1.98 × 10^−8^) [20], is an intronic SNP located on chromosome 22q13 between two genes, *C1QTNF6* (C1q and tumor necrosis factor related protein 6) and *SSTR3 *(somatostatin receptor 3). We found that *C1QTNF6* (rs229541) is associated with development of IA but not associated with progression to T1D in IA positive children, suggesting that this gene may play a role early in the development of T1D related to the initial appearance of autoimmunity.

SNP (rs10517086) exhibits an age-related effect with development of IA, with an increased risk of developing IA before the age of two or in younger ages, and a null effect in older ages. Children carrying a risk allele for SNP (rs10517086) developed IA significantly earlier than children without a risk allele. Based on these epidemiologic analyses, future studies should investigate mechanisms as to how this SNP influences risk of early autoimmunity. SNP (rs10517086), which was first discovered through another GWAS and meta-analysis of T1D, is located within a gene desert on chromosome 4. Loci in or near genes without a known function or in regions not containing annotated genes may indicate involvement of long-range gene expression regulatory elements and/or nonprotein-coding RNA genes [26].

*UBASH3A* (rs3788013) was associated with development of T1D, but not with either of the stages preceding this (development of IA and progression from IA to T1D). *UBASH3A* (rs3788013) is an intronic SNP located on chromosome 21q22 in the *UBASH3A *(ubiquitin associated and SH3 domain containing A) gene. *UBASH3A* is expressed predominantly in T cells suppressing T-cell receptor signaling [27]. *UBASH3A* has also been associated with other autoimmune diseases, such as celiac disease and rheumatoid arthritis [28]. Another *UBASH3A* SNP, *UBASH3A *(rs11203203), was found to be associated with both development of IA and development of T1D in a previous DAISY analysis [11]. The LD between *UBASH3A* (rs3788013) and *UBASH3A* (rs11203203) is *r* ^2^ = 0.491 and *D'* = 0.801. DAISY uses two definitions of IA, one that defines IA as the presence of at least one islet autoantibody on two consecutive visits (which is the definition used in the present analysis) and the other that further requires that the children still be autoantibody positive or diabetic on their most recent visit (which is the definition used in the previous analysis). The definition used in the previous study is closer to T1D, which makes our *UBASH3A* (rs3788013) association with T1D consistent with what was previously found with *UBASH3A* (rs11203203).

In combination with those presented in this paper, DAISY has now investigated 34 non-HLA T1D candidate SNPs for association with development of IA, progression from IA to T1D, and/or development of T1D with multiple examples of stage-related heterogeneity, which are presented in Table 3. Investigating 20 non-HLA SNPs for development of IA, progression from IA to T1D, and/or development of T1D, Steck et al. found *PTPN22* (rs2476601) associated with development of IA, but not progression from IA to T1D, and *CTLA4* (rs231775) associated with progression from IA to T1D, but not development of IA [9]. *PTPN2* (rs1893217) was only associated with development of IA, while *UBASH3A* (rs11203203) was associated with development of IA and development of T1D [11]. *INS* (rs689) was not associated with development of IA nor progression from IA to T1D but was associated with development of T1D [9, 11]. *PTPN22* (rs2476601) was also associated with development of T1D [11]. Here we investigated fourteen additional non-HLA T1D candidate SNPs and found *C1QTNF6* (rs229541) associated with development of IA and development of T1D, but not progression from IA to T1D. *UBASH3A* (rs3788013) was associated with development of T1D, but not with either of the stages preceding this (development of IA and progression from IA to T1D) and SNP (rs10517086) was associated with development of T1D, while exhibiting an age-related effect with IA risk but was not associated with progression from IA to T1D. The SNPs investigated in these three studies are summarized in Table 3. The distinction between the risk factors for islet autoimmunity versus progression to type 1 diabetes in IA positive children is important because it may allow us to explore potentially different mechanisms of *triggering* islet autoimmunity versus *epitope spreading and progressive loss of beta-cell mass* leading to overt diabetes.

GWAS are important for identifying new candidate regions associated with a clinical outcome, such as T1D, in a large number of cases and controls. Prospective birth cohort studies, like DAISY, are then able to take these newly identified candidate regions and look for associations with different stages in the disease process and at different ages. As a prospective birth cohort study following children at increased risk for developing T1D from birth, we are able to capture the preclinical phase of T1D, islet autoimmunity. This allows us to study two separate stages in the natural history of T1D: development of IA and progression to T1D in IA positive children. We are also able to study whether certain exposures are important at one age, but not another.

Due to the cost associated with following a large group of children from birth into adulthood, our sample sizes are much smaller than those obtained for GWAS. Our lack of association for many of these SNPs is not evidence against their association with T1D but may result from limited power, especially in the progression from IA to T1D stage. We also have a very unique population comprised of children with high risk HLA genotypes and it is possible that the effect of these SNPs differs based on one's HLA risk status. The risk for non-HLA loci appears to be lower in individuals carrying high-risk HLA genotypes, as has been seen with *PTPN22* (rs2476601) [21, 29] and *TCF7* (rs5742913) [30, 31].

We believe that taking these GWAS identified candidate regions and studying them in the context of the natural history of T1D are central to better understanding the disease process and where in the disease process genetic loci may be important. This will allow us to create more accurate risk prediction models for both stages in the natural history of the disease, as well as inform the design of targeted interventions to prevent or slow the progression of IA and subsequent development of T1D.

## 5. Conclusions

The effect of a SNP may act nonlinearly, with an effect at early ages but not later ages or vice versa. Our results provide evidence that SNP (rs10517086) is acting on early risk of IA, with the age at onset of IA occurring significantly earlier in children with a minor allele compared to children with no minor alleles. By ignoring heterogeneity in the etiology of disease, valuable associations may be missed that could aid in better understanding complex diseases, such as T1D.

## Supplementary Material

Unadjusted SNP association analyses are presented in Supplemental Table 1. In the unadjusted SNP analyses, C1QTNF6 (rs229541) was associated with development of IA. None of the SNPs were associated with progression to T1D in IA positive children. Four SNPs, C1QTNF6 (rs229541), IL2RA (rs12722563), UBASH3A (rs3788013), and SNP (rs10517086) were associated with development of T1D.

## Figures and Tables

**Table 1:** Demographic characteristics of DAISY non-Hispanic white population.

Characteristic	Development of islet autoimmunity (IA) (*n* = 1619)				Progression from IA to type 1 diabetes (T1D) (*n* = 132)			
	Children positive for IA (*n* = 117)	Children negative for IA (*n* = 1502)	Univariate HR and 95% CI	*P* value	IA positive children who progressed to T1D (*n* = 50)	IA positive children who have not progressed to T1D (*n* = 82)	Univariate HR and 95% CI	*P* value
Mean age (years)	6.2 ± 4.2^a^	9.9 ± 5.7^b^	N/A	N/A	8.7 ± 3.9^c^	14.1 ± 4.2^b^	N/A	N/A
Mean age at first IA positive visit (years)	N/A	N/A	N/A	N/A	3.9 ± 2.9	7.2 ± 4.2	0.86 (0.77, 0.95)	0.003
HLA-DR3/4, DQB1*0302	43 (36.8%)	248 (16.5%)	2.97 (2.01, 4.39)	<0.0001	28 (56.0%)	19 (23.2%)	2.79 (1.65, 4.72)	0.0001
First degree relative with T1D	75 (64.1%)	737 (49.1%)	1.41 (0.96, 2.07)	0.08	36 (72.0%)	54 (65.9%)	1.02 (0.56, 1.85)	0.96
Sex (female)	62 (53.0%)	712 (47.4%)	1.22 (0.85, 1.74)	0.28	24 (48.0%)	44 (53.7%)	1.03 (0.59, 1.83)	0.91

CI: confidence interval; DAISY: Diabetes Autoimmunity Study in the Young; HLA: human leukocyte antigen; HR: hazard ratio; IA: islet autoimmunity; T1D: type 1 diabetes.

^a^Age at first IA positive visit.

^b^Age at last followup.

^c^Age at T1D diagnosis.

**Table 2:** Association between non-HLA T1D candidate SNPs and development of IA, progression from IA to T1D, and development of T1D adjusted for HLA-DR3/4, DQB1*0302 genotype and first degree relative with T1D.

SNP	Minor allele	Development of IA (*n* = 1619)			Progression from IA to T1D (*n* = 132)		Development of T1D (*n* = 1619)	
		MAF^a^	Adjusted HR^b^ and 95% CI	*P* value	Adjusted HR^c^ and 95% CI	*P* value	Adjusted HR^b^ and 95% CI	*P* value
*C1QTNF6* (rs229541)	A	0.44	**1.57 (1.20, 2.05**)^**d**^	**0.001**	1.13 (0.75, 1.71)^d^	0.56	**1.95 (1.33, 2.87**)^**d**^	**0.001**
*C6orf173* (rs9388489)	G	0.46	1.15 (0.88, 1.51)^d^	0.31	0.87 (0.61, 1.24)^d^	0.44	1.03 (0.69, 1.54)^d^	0.87
*C14orf181* (rs1465788)	T	0.28	0.97 (0.73, 1.27)^d^	0.80	1.09 (0.62, 1.92)^e^	0.77	0.70 (0.40, 1.23)^e^	0.21
*IL2* (rs2069762)	C	0.29	1.08 (0.82, 1.43)^d^	0.58	1.22 (0.79, 1.87)^d^	0.38	1.20 (0.79, 1.83)^d^	0.40
*IL2* (rs4505848)	G	0.35	1.04 (0.80, 1.34)^d^	0.78	0.93 (0.67, 1.28)^d^	0.65	1.18 (0.82, 1.70)^d^	0.37
*IL2RA* (rs12722563)	A	0.12	0.90 (0.58, 1.41)^e^	0.65	0.52 (0.17, 1.57)^e^	0.25	0.43 (0.17, 1.08)^e^	0.07
*IL2RA* (rs2104286)	C	0.27	0.85 (0.63, 1.15)^d^	0.28	1.26 (0.70, 2.27)^e^	0.44	0.77 (0.43, 1.38)^e^	0.38
*IL7R* (rs6897932)	T	0.28	0.93 (0.68, 1.28)^d^	0.66	0.86 (0.49, 1.53)^e^	0.61	0.88 (0.50, 1.55)^e^	0.65
*PRKCQ* (rs947474)	G	0.17	1.19 (0.80, 1.77)^e^	0.40	0.76 (0.41, 1.42)^e^	0.39	0.87 (0.47, 1.64)^e^	0.67
*SKAP2* (rs7804356)	C	0.25	0.90 (0.66, 1.22)^d^	0.49	1.54 (0.83, 2.85)^e^	0.17	1.01 (0.57, 1.78)^e^	0.99
*SMARCE1* (rs7221109)	T	0.35	0.94 (0.72, 1.22)^d^	0.63	0.94 (0.57, 1.57)^d^	0.82	0.73 (0.45, 1.20)^d^	0.21
*TLR8* (rs5979785)	C	0.20	0.82 (0.64, 1.05)^d^	0.11	0.84 (0.61, 1.16)^d^	0.29	0.86 (0.60, 1.24)^d^	0.43
*UBASH3A* (rs3788013)	A	0.44	1.19 (0.89, 1.59)^d^	0.25	1.03 (0.72, 1.48)^d^	0.86	**1.63 (1.04, 2.54**)^**d**^	**0.03**
rs10517086	A	0.30	∗	∗	1.36 (0.77, 2.41)^e^	0.30	**2.03 (1.35, 3.03**)^**d**^	**0.001**

CI: confidence interval; DAISY: Diabetes Autoimmunity Study in the Young; HLA: human leukocyte antigen; HR: hazard ratio; IA: islet autoimmunity; MAF: minor allele frequency; T1D: type 1 diabetes.

^a^Minor allele frequency (MAF) calculated for children negative for IA.

^b^Adjusted for HLA-DR3/4, DQB1*0302 genotype and first degree relative with T1D. *TLR8* (rs5979785) is additionally adjusted for sex because it is on the X chromosome.

^c^Adjusted for HLA-DR3/4, DQB1*0302 genotype, first degree relative with T1D, and age at first antibody positive visit. *TLR8* (rs5979785) is additionally adjusted for sex because it is on the X chromosome.

^d^SNP analyzed additively with HR representing increase in risk for each additional minor allele.

^e^SNP analyzed dichotomously with HR representing increase in risk for at least one minor allele.

*SNP rs10517086 did not meet the assumptions of proportional hazards in the development of IA analysis and therefore was modeled using a restricted cubic spline (Figure 1).

**Table 3:** Non-HLA T1D candidate SNPs associated with development of IA, progression from IA to T1D, and/or development of T1D in DAISY.

SNP	Development of IA		Progression from IA to T1D		Development of T1D	
	Adjusted HR and 95% CI	*P* value	Adjusted HR and 95% CI	*P* value	Adjusted HR and 95% CI	*P* value
*C1QTNF6* (rs229541)	**1.57 (1.20, 2.05**)^**abc**^	**0.001**	1.13 (0.75, 1.71)^abd^	0.56	**1.95 (1.33, 2.87**)^**abc**^	**0.001**
*CTLA4 *(rs231775)	1.12 (0.86, 1.46)^aef^	0.42	**0.54 (0.33, 0.88**)^**aeg**^	**0.01**	1.00 (0.70, 1.43)^ahc^	1.00
*INS* (rs689)	1.39 (0.99, 1.95)^aef^	0.05	1.34 (0.72, 2.52)^aeg^	0.35	**1.75 (1.08, 2.83**)^**ahc**^	**0.02**
*PTPN2 *(rs1893217)	**1.42 (1.02, 1.99**)^**ach**^	**0.04**	0.65 (0.27, 1.60)^ijk^	0.35	0.99 (0.60, 1.66)^ach^	0.98
*PTPN22* (rs2476601)	**1.83 (1.27, 2.63**)^**aef**^	**0.001**	0.98 (0.50, 1.93)^aeg^	0.96	**1.74 (1.04, 2.90**)^**ahc**^	**0.03**
*UBASH3A *(rs11203203)	**1.46 (1.11, 1.91**)^**ach**^	**0.01**	∗∗	∗∗	**1.83 (1.28, 2.64**)^**ach**^	**0.001**
*UBASH3A* (rs3788013)	1.19 (0.89, 1.59)^abc^	0.25	1.03 (0.72, 1.48)^abd^	0.86	**1.63 (1.04, 2.54**)^**abc**^	**0.03**
rs10517086	∗	∗	1.36 (0.77, 2.41)^bdj^	0.30	**2.03 (1.35, 3.03**)^**abc**^	**0.001**

DAISY: Diabetes Autoimmunity Study in the Young; HLA: human leukocyte antigen; HR: hazard ratio; CI: confidence interval; IA: islet autoimmunity; T1D: type 1 diabetes.

^a^SNP analyzed additively with HR representing increase in risk for each additional minor allele.

^b^Listed in Table 2 of the current paper.

^c^Adjusted for HLA-DR3/4, DQB1*0302 genotype and first degree relative with T1D.

^d^Adjusted for HLA-DR3/4, DQB1*0302 genotype, first degree relative with T1D, and age at first antibody positive visit.

^e^From Steck et al. (2009) [9].

^f^Adjusted for HLA-DR3/4, DQB1*0302 genotype, ethnicity, sex, and first degree relative with type 1 diabetes.

^g^Adjusted for HLA-DR3/4, DQB1*0302 genotype, ethnicity, sex, first degree relative with type 1 diabetes, and age at first antibody positive visit.

^h^From Steck et al.(2012) [11].

^i^From Frederiksen et al. (2013) [12]. Frederiksen et al. (2013) [12] did not find an association between *PTPN2* (rs1893217) and development of IA, which can be attributed to the use of different IA case definitions used in the manuscripts by Steck et al. (2009) [9] and Frederiksen et al. (2013) [12].

^j^SNP analyzed dichotomously with HR representing increase in risk for at least one minor allele.

^k^Adjusted for *PTPN2* (rs478582), HLA-DR3/4, DQB1*0302 genotype, first degree relative with type 1 diabetes, ethnicity, and age at first IA positive visit.

**Analysis not conducted.

*SNP rs10517086 did not meet the assumptions of proportional hazards in the development of IA analysis and therefore was modeled using a restricted cubic spline (Figure 1 of the current paper).

## Abbreviations

CI: Confidence interval
C1QTNF6: C1q and tumor necrosis factor related protein 6
C6orf173: Chromosome 6 open reading frame 173
C14orf181: Chromosome 14 open reading frame 181
CTLA4: Cytotoxic T-lymphocyte-associated protein 4
HLA: Human leukocyte antigen
HR: Hazard ratio
IA: Islet autoimmunity
IFIH1: Interferon induced with helicase C domain 1
IL2: Interleukin 2
IL2RA: Interleukin 2 receptor, alpha
IL7R: Interleukin 7 receptor
INS: Insulin
PRKCQ: Protein kinase C, theta
PTPN22: Protein tyrosine phosphatase, nonreceptor type 22
SKAP2: Src kinase associated phosphoprotein
SMARCE1: SWI/SNF related, matrix associated, actin dependent regulator of chromatin, subfamily E, member 1
SNP: Single nucleotide polymorphism
T1D: Type 1 diabetes
TLR8: Toll-like receptor 8
UBASH3A: Ubiquitin associated and SH3 domain containing A.
