# Supplementary material for: Evidence of Stage- and Age-Related Heterogeneity of Non-HLA SNPs and Risk of Islet Autoimmunity and Type 1 Diabetes: The Diabetes Autoimmunity Study in the Young
Source: Clin Dev Immunol. 2013 Dec 4;2013:417657. doi: 10.1155/2013/417657 (PMC3866813; doi:10.1155/2013/417657)
Supplement: Supplementary file 1 — Unadjusted SNP association analyses are presented in Supplemental Table 1. In the unadjusted SNP analyses, C1QTNF6 (rs229541) was associated with development of IA. None of the SNPs were associated with progression to T1D in IA positive children. Four SNPs, C1QTNF6 (rs229541), IL2RA (rs12722563), UBASH3A (rs3788013), and SNP (rs10517086) were associated with development of T1D. [file 417657.f1.pdf]

Supplemental Table 1: Association between Non-HLA T1D Candidate SNPs and Development of IA, Progression from IA to T1D, and Development of T1D Unadjusted for HLA-DR3/4,DQB1*0302 and First Degree Relative with T1D.

	Development of IA (n = 1619)		Progression from IA to T1D (n = 132)		Development of T1D (n = 1619)	
SNP	HR and 95% CI	*P* Value	HR and 95% CI[a]	*P* Value	HR and 95% CI	*P* Value
C1QTNF6 (rs229541)	**1.55 (1.18, 2.02)[b]**	**0.001**	1.25 (0.86, 1.83)[b]	0.25	**1.94 (1.33, 2.84)[b]**	**0.001**
C6orf173 (rs9388489)	1.16 (0.88, 1.52)[b]	0.29	0.93 (0.68, 1.27)[b]	0.66	1.05 (0.70, 1.56)[b]	0.83
C14orf181 (rs1465788)	1.00 (0.77, 1.31)[b]	0.98	1.04 (0.59, 1.84)[c]	0.89	0.79 (0.46, 1.39)[c]	0.42
IL2 (rs2069762)	1.06 (0.80, 1.40)[b]	0.69	1.33 (0.88, 2.00)[b]	0.18	1.19 (0.78, 1.81)[b]	0.42
IL2 (rs4505848)	1.02 (0.79, 1.32)[b]	0.88	0.93 (0.66, 1.30)[b]	0.66	1.16 (0.80, 1.68)[b]	0.43
IL2RA (rs12722563)	0.83 (0.53, 1.30)[c]	0.41	0.63 (0.23, 1.72)[c]	0.37	**0.37 (0.15, 0.92)[c]**	**0.03**
IL2RA (rs2104286)	0.82 (0.60, 1.10)[b]	0.19	1.04 (0.59, 1.83)[c]	0.90	0.66 (0.37, 1.19)[c]	0.17
IL7R (rs6897932)	0.93 (0.68, 1.29)[b]	0.67	0.88 (0.51, 1.51)[c]	0.64	0.84 (0.48, 1.49)[c]	0.55
PRKCQ (rs947474)	1.11 (0.74, 1.65)[c]	0.62	0.64 (0.36, 1.12)[c]	0.12	0.75 (0.40, 1.42)[c]	0.38
SKAP2 (rs7804356)	0.90 (0.66, 1.22)[b]	0.49	1.32 (0.78, 2.24)[c]	0.31	0.96 (0.54, 1.69)[c]	0.88
SMARCE1 (rs7221109)	0.94 (0.71, 1.23)[b]	0.63	0.88 (0.51, 1.51)[b]	0.63	0.69 (0.41, 1.16)[b]	0.16
TLR8 (rs5979785)[d]	0.84 (0.64, 1.09)[b]	0.18	0.90 (0.65, 1.26)[b]	0.54	0.92 (0.63, 1.34)[b]	0.66
UBASH3A (rs3788013)	1.22 (0.92, 1.63)[b]	0.17	1.28 (0.88, 1.87)[b]	0.20	**1.76 (1.15, 2.69)[b]**	**0.01**
rs10517086	*	*	1.60 (0.89, 2.87)[c]	0.12	**1.90 (1.28, 2.83)[b]**	**0.002**

Abbreviations: CI, confidence interval; DAISY, Diabetes Autoimmunity Study in the Young; HLA, human leukocyte antigen; HR, hazard ratio; IA, islet autoimmunity; MAF, minor allele frequency; T1D, type 1 diabetes.

[a]Adjusted for age at first autoantibody positive visit.

[b]SNP analyzed additively with HR representing increase in risk for each additional minor allele.

[c]SNP analyzed dichotomously with HR representing increase in risk for at least one minor allele.

[d]*TLR8* (rs5979785) is adjusted for sex because it is on the X chromosome.

*SNP rs10517086 did not meet the assumptions of proportional hazards in the development of IA analysis.
